# Supplementary material for: Visual art inspired by climate change—An analysis of audience reactions to 37 artworks presented during 21st UN climate summit in Paris
Source: PLoS One. 2021 Feb 19;16(2):e0247331. doi: 10.1371/journal.pone.0247331 (PMC7894892; doi:10.1371/journal.pone.0247331)
Supplement: S1 Table — (DOCX) [file pone.0247331.s001.docx]

**S1 Table. List of artworks st.**

| Number | Original name | English name | participants surveyed |
| --- | --- | --- | --- |
| 1 | Our vision for the future | Our vision for the future | 30 |
| 2 | Honeybees | Honeybees | 22 |
| 3 | Artificial Wonderland - Disorder | Artificial Wonderland - Disorder | 26 |
| 4 | Still life - Disorder C | Still life - Disorder C | 23 |
| 5 | Breaking the surface | Breaking the surface | 30 |
| 6 | Climate on the wall | Climate on the wall | 15 |
| 7 | Fridge Cube | Fridge Cube | 16 |
| 8 | Crystal Ball | Crystal Ball | 22 |
| 9 | Honey Roads | Honey Roads | 20 |
| 10 | Climats artificiel exhibition |  | 38 |
| 11 | Le film noir de Lampedusa |  | 26 |
| 12 | Nervous Trees | Nervous Trees | 19 |
| 13 | Ribbontree | Ribbontree | 15 |
| 14 | Exit | Exit | 20 |
| 15 | Unbearable | Unbearable | 32 |
| 16 | Mur vegetal |  | 20 |
| 17 | Whale/Balleine Blue | Whale/Balleine Blue | 22 |
| 18 | Kiss Kiss Game | Kiss Kiss Game | 23 |
| 19 | Nouveau Monde | New World | 31 |
| 20 | Oeuvre Ensemble pour le climat |  | 20 |
| 21 | Veolia | Veolia | 30 |
| 22 | Disorder Drowing World | Disorder Drowing World | 20 |
| 23 | Antarctica World Passport Delivery Bureau | Antarctica World Passport Delivery Bureau | 33 |
| 24 | Il était une fois… demain |  | 20 |
| 25 | Cloudscapes | Cloudscapes | 24 |
| 26 | Act responsible | Act responsible | 21 |
| 27 | Stoves | Stoves | 37 |
| 28 | Ice watch | Ice watch | 33 |
| 29 | Climat, état d´urgence |  | 21 |
| 30 | Pachamama | Pachamama | 22 |
| 31 | Venus of trash isle | Venus of trash isle | 21 |
| 32 | Amrita/Manthan - Gujarat women empowerment | Amrita/Manthan - Gujarat women empowerment | 21 |
| 33 | Gaia | Gaia | 22 |
| 34 | Birdman/Dreams/Redemption | Birdman/Dreams/Redemption | 23 |
| 35 | Sertella Septentrionalis/ Gorgonia Ventalina |  | 19 |
| 36 | Mushroom Iceberg/Artic Ice 4 | Mushroom Iceberg/Artic Ice 4 | 25 |
| 37 | La Terre | The Earth | 21 |
