# Supplementary material for: Visual art inspired by climate change—An analysis of audience reactions to 37 artworks presented during 21st UN climate summit in Paris
Source: PLoS One. 2021 Feb 19;16(2):e0247331. doi: 10.1371/journal.pone.0247331 (PMC7894892; doi:10.1371/journal.pone.0247331)
Supplement: S2 Table — CR = composite reliability; AVE = average variance extracted; B = unstandardized loading; SE = standard error; Beta = standardized loading; RES = unstandardized unexplained residual variance; p = significance level of loading. (DOCX) [file pone.0247331.s002.docx]

**S2 Table. Loadings on the latent variables in the measurement model (with "identification with the artist" included in the upper half, with "identification with the artist" excluded in the lower half of the table).** CR = composite reliability; AVE = average variance extracted; B = unstandardized loading; SE = standard error; Beta = standardized loading; RES = unstandardized unexplained residual variance; p = significance level of loading.

| Latent var. | Items loading on the latent variable | B | SE | Beta | RES | p |
| --- | --- | --- | --- | --- | --- | --- |
| Positive emotions (CR=.846; AVE=.647) | |  |  |  |  |  |
|  | Happy | 1.000 | - | .762 | .419 | - |
|  | Hope | 1.113 | .036 | .849 | .279 | <.001 |
|  | Inspiration | 1.046 | .031 | .797 | .365 | <.001 |
|  |  |  |  |  |  |  |
| Negative emotions (CR=.809; AVE=.590) | |  |  |  |  |  |
|  | Anger | 1.000 | - | .868 | .246 | - |
|  | Anxiety | .865 | .035 | .751 | .437 | <.001 |
|  | Sadness | .759 | .034 | .659 | .566 | <.001 |
|  |  |  |  |  |  |  |
| Identification with the artist (CR=.596; AVE=.345) | |  |  |  |  |  |
|  | S/he is someone like me | 1.000 | - | .466 | .783 | - |
|  | S/he shares similar values | 1.632 | .133 | .760 | .422 | <.001 |
|  | S/he reflects the values of the public | 1.001 | .107 | .466 | .783 | <.001 |
|  |  |  |  |  |  |  |
| Reflections on the artwork (CR=.910; AVE=.635) | |  |  |  |  |  |
|  | The work made me reflect | 1.000 | - | .668 | .554 | - |
|  | The work is relevant for daily life | 1.013 | .037 | .676 | .543 | <.001 |
|  | The work made me reflect consequences | 1.089 | .035 | .727 | .472 | <.001 |
|  | Climate change risk perception | 1.260 | .047 | .841 | .292 | <.001 |
|  | The work made me reflect on my role | 1.384 | .054 | .924 | .146 | <.001 |
|  | The work had a personal impact | 1.266 | .053 | .845 | .285 | <.001 |
| Positive emotions (CR=.846; AVE=.647) | |  |  |  |  |  |
|  | Happy | 1.000 | - | .766 | .413 | - |
|  | Hope | 1.100 | .035 | .842 | .290 | <.001 |
|  | Inspiration | 1.045 | .031 | .800 | .360 | <.001 |
|  |  |  |  |  |  |  |
| Negative emotions (CR=.809; AVE=.589) | |  |  |  |  |  |
|  | Anger | 1.000 | - | .867 | .248 | - |
|  | Anxiety | .859 | .035 | .753 | .434 | <.001 |
|  | Sadness | .758 | .034 | .657 | .568 | <.001 |
|  |  |  |  |  |  |  |
| Reflections on the artwork (CR=.909; AVE=.631) | |  |  |  |  |  |
|  | The work made me reflect | 1.000 | - | .662 | .562 | - |
|  | The work is relevant for daily life | .996 | .037 | .659 | .566 | <.001 |
|  | The work made me reflect consequences | 1.098 | .035 | .726 | .473 | <.001 |
|  | Climate change risk perception | 1.273 | .050 | .842 | .290 | <.001 |
|  | The work made me reflect on my role | 1.402 | .057 | .927 | .140 | <.001 |
|  | The work had a personal impact | 1.250 | .055 | .847 | .282 | <.001 |
