# Supplementary material for: Visual art inspired by climate change—An analysis of audience reactions to 37 artworks presented during 21st UN climate summit in Paris
Source: PLoS One. 2021 Feb 19;16(2):e0247331. doi: 10.1371/journal.pone.0247331 (PMC7894892; doi:10.1371/journal.pone.0247331)
Supplement: S3 Table — The table displays Pearson correlations; *** p < .001, ** p < .01, * p < .05. (DOCX) [file pone.0247331.s003.docx]

**S3 Table. Correlations of all latent variables and single-item measures in the measurement model (model with "identification with the artist" below the diagonal; model without "identification with the artist" above the diagonal).** The table displays Pearson correlations; *** p<.001, ** p<.01, * p<.05

|  | 1 | 2 | 3 | 4 | 5 | 6 | 7 | 8 |
| --- | --- | --- | --- | --- | --- | --- | --- | --- |
| 1 pos emo |  | -.208 *** | .275 *** | - | .391 *** | .126  ** | .185 *** | .023 |
| 2 neg emo | -.208 *** |  | .094  * | - | .422 *** | .190 *** | .038 | .004 |
| 3 quality | .275 *** | .094  * |  | - | .450 *** | .164 *** | .117  ** | -.081  * |
| 4 ident artist | .395 *** | .094 | .445 *** |  | - | - | - | - |
| 5 reflect | .391 *** | .422 *** | .450 *** | .588 *** |  | .330 *** | .170 *** | -.123 ** |
| 6 policy support | .126  ** | .190 *** | .164 *** | .368 *** | .329 *** |  | .442 *** | -.046 |
| 7 env. attitude | .185 *** | .036 | .117  ** | .311 *** | .170 *** | .442 *** |  | -.017 |
| 8 gender (1= male/0=female) | .023 | .004 | -.081 ** | -.110 * | -.123 ** | -.046 | -.017 |  |
