# Supplementary material for: Visual art inspired by climate change—An analysis of audience reactions to 37 artworks presented during 21st UN climate summit in Paris
Source: PLoS One. 2021 Feb 19;16(2):e0247331. doi: 10.1371/journal.pone.0247331 (PMC7894892; doi:10.1371/journal.pone.0247331)
Supplement: S4 Table — As the outcome variable "support for climate policy" was ordered-categorical, we report the three thresholds in the table. The outcome variable was linked in the regression through a probit link. B = unstandardized loading; SE = standard error; Beta = standardized loading; p = significance level. (DOCX) [file pone.0247331.s004.docx]

**S4 Table. A simplified model to test the interaction between environmental attitude and reflection on the artwork (with gender as a control variable).** As the outcome variable "support for climate policy" was ordered-categorical, we report the three thresholds in the table. The outcome variable was linked in the regression through a probit link. B = unstandardized loading; SE = standard error; Beta = standardized loading; p = significance level.

|  | B | SE | Beta | p | R^2^ |
| --- | --- | --- | --- | --- | --- |
| Threshold 1 | -2.858 | .181 | -2.470 | <.001 |  |
| Threshold 2 | -2.090 | .101 | -1.807 | <.001 |  |
| Threshold 3 | -.603 | .079 | -.522 | <.001 |  |
|  |  |  |  |  |  |
| Reflection on the artwork | .028 | .005 | .243 | <.001 |  |
| Environmental attitude | .383 | .050 | .396 | <.001 |  |
| Reflection on the artwork * environmental attitude | -.007 | .004 | -.083 | .034 |  |
| Gender (1=male / 0 = female) | .034 | .117 | .015 | .771 |  |
|  |  |  |  |  | .253 |
