# Supplementary material for: Visual art inspired by climate change—An analysis of audience reactions to 37 artworks presented during 21st UN climate summit in Paris
Source: PLoS One. 2021 Feb 19;16(2):e0247331. doi: 10.1371/journal.pone.0247331 (PMC7894892; doi:10.1371/journal.pone.0247331)
Supplement: Attachment — Submitted filename: Response to Reviewers.docx [file pone.0247331.s005.docx]

# Response to Reviewer

Dear editor and reviewer,

We like to express our gratitude for your thoughtful inputs that have improved the manuscript. Please see the table below for all changes made to the manuscript based on your feedback.

| Editor’s comments | Actions taken |
| --- | --- |
| After careful consideration, we feel that it has merit but does not fully meet PLOS ONE’s publication criteria as it currently stands. Therefore, we invite you to submit a revised version of the manuscript that addresses the points raised during the review process. | Thank you for the considerate comments to the manuscript which led to a thorough revision. We feek that they led to an improvement of the manuscript. |
| Please submit your revised manuscript by Jan 28 2021 11:59PM. If you will need more time than this to complete your revisions, please reply to this message or contact the journal office at [plosone@plos.org](mailto:plosone@plos.org). | We sent the revision within the given time frame. |
| Please include the following items when submitting your revised manuscript:   - A rebuttal letter that responds to each point raised by the academic editor and reviewer(s). You should upload this letter as a separate file labeled 'Response to Reviewers'. - A marked-up copy of your manuscript that highlights changes made to the original version. You should upload this as a separate file labeled 'Revised Manuscript with Track Changes'. - An unmarked version of your revised paper without tracked changes. You should upload this as a separate file labeled 'Manuscript'. | This table with a detailed description of the changes made based on the input from the editor and the reviewer has been uploaded as a separate file to the system as “response to reviewers”.  A version of the manuscript with marked-up changes and an unmarked version have been uploaded with the names suggested here. |
| Please ensure that your manuscript meets PLOS ONE's style requirements, including those for file naming. The PLOS ONE style templates can be found at  <https://journals.plos.org/plosone/s/file?id=wjVg/PLOSOne_formatting_sample_main_body.pdf> and  <https://journals.plos.org/plosone/s/file?id=ba62/PLOSOne_formatting_sample_title_authors_affiliations.pdf> | The main manuscript body and the title page have been adapted to PLOS ONE’s style requirements. |
| 2. Please modify the title to ensure that it is meeting PLOS’ guidelines (<https://journals.plos.org/plosone/s/submission-guidelines#loc-title>). In particular, the title should be "specific, descriptive, concise, and comprehensible to readers outside the field" and in this case it is not informative and specific about your study's scope and methodology. | We changed the title of the manuscript according to the suggestions. It is now “Visual art inspired by climate change – An analysis of audience reactions to 37 artworks presented during 21st UN climate summit in Paris”.  However, we allow us a small comment here: In another review process for a different journal, we were just recently strongly recommended ny the editor to find a more “catchy” title as they tend to attract more attention to papers. We believe that our original title would have qualified for being “more catchy”. We also believe that it actually reflected one of the main points of our analysis, namely that climate communication through artworks adds an emotional dimension to climate change communication. However, as different journals have different styles, we made the title of the manuscript more “ordinary” to fit in with the other PLOS ONE titles. |
| I have now collected one review from one expert in the field. I was unable to find a second reviewer, but the review I could collect is very detailed and helpful. Therefore, I feel confident in making a decision with only one review. The reviewer likes the paper but suggests several improvements. Therefore, I would like to invite you to revise your manuscript following the reviewer's comments. I am looking forward for the revision. | Thank you for making a decision with only one review. We agree that the review is detailed and balanced and we address all comments by the reviewer in the revision (as outlined in the table below). |

| Reviewer’s comments | Actions taken |
| --- | --- |
| This is a well-written paper that describes an interesting, novel study about an understudied topic--the relationship between art viewing and various emotional and psychological responses. As this is an understudied area, this study has potential to add to this specific field. | Dear reviewer, thank you for seeing the potential in our study. |
| However, I have significant questions about some aspects of the statistical analyses (especially the factor analysis). | Please see our responses to the detailed comments below. |
| Additionally, many of the claims articulated here go further than merited by the data and rely on several assumptions. | We carefully went through the manuscript and rephrased cases where the original sentences implied interpretations which were not merited by the (correlational) data. |
| While I believe this study does have something to offer in regards to climate-related art viewing, more work needs to be done to clarify the validity of this study and to temper its claims. | We hope that our revised version of the manuscript satisfies the reviewer’s standards. |
| The factor analysis is unclear and lacking key information. Specifically, the authors refer to Table B that is supposed to show loadings on latent variables. While it shows loading of each item, it is unclear what latent variables each is loading on, as well as each item’s unique loading. It is also unclear what percentage of variability in the CFA model is explained by each latent variable. This is an important aspect of accepting the overall results, as analysis relies on these groupings of items as variables and builds the SEM on these groupings. In particular, I have questions about the “Reflection” variable, which seems like it is being measured by quite different items. It is hard to believe that they all load uniquely onto one latent variable, and I would like to see clearer evidence for that before fully trusting the SEM. | Part of the information the reviewer is asking for was already in the table now labeled “S2 Table” (the latent variable each item is loading on is named before the arrow, the unique loading is the B [unstandardized] and beta [standardized]).  We added the remaining requested information to the table and restructured the table to be easier to read as well as added a legend.  We agree that the “Reflection” variable is combining rather diverse items. However, the model fit indicates that assuming unidimensionality for that latent variable (and the other latent variables) seems to lead to satisfactory fit to the data. Furthermore, the detailed analysis of the measurement model indicates a good reliability of this variable (composite reliability is .910 and the average variance extracted is .635). Following up on that note: The latent variable “identification with the artist” displays more challenges than the “reflection” variable. Composite reliability and AVE are rather low and the AVE is at about the same level as the square of the largest correlation between the latent variables. We considered replacing the latent variable with a one item measure of identification with the artist (“S/he shares similar values”), but decided against it because we consider even a relatively poor latent variable better than a one-item measure where we have no control about the measurement error. We mention this consideration in the paper now. |
| I also have questions about some aspects of the SEM and would like further clarification. For instance, on page 9, the authors say: “The data has a clustered two-level structure, since the audience responses have been recorded at 37 venues for artworks in Paris.” What levels are you clustering the data at? For venue? Or for personal characteristics? What two levels constitute the structure? | Yes, the data is clustered at the venue level. To be clear here: This is not a cluster analysis (which seems to be implied by the reviewer’s question about “personal characteristics” as a clustering criterion) but a multilevel-SEM where the clusters are defined by a second order structure (here attendants of the same venue). To estimate standard errors correctly and to be able to assess the artwork level-effects simulationsly with the person-level effects, this clustered (also referred to as nested) data structure needs to be acknowledged in the analysis. Technically, the analysis algorithm is separating variance in dependent variables into a between-artworks part and a within-artworks (hence, between persons visiting the same artwork) part. We slightly rephrased the sentence to get this out more clearly.  See for example here for an introduction to multilevel-SEM: Rabe-Hesketh, S., Skrondal, A., & Zheng, X. (2007). Multilevel structural equation modeling. In Handbook of latent variable and related models (pp. 209-227). North-Holland. |
| Is there any error measurement associated with calculation of latent variable scores for each person? Using scores instead of variables could introduce more error, I would think. | The latent variable scores were assessed in the estimation of the model, not posthoc as sum or mean scores. An assumption in SEM is that the latent variables are free from measurement error because they only reflect the meaningful variance shared between the indicators (which have a measurement error component – more technically, they have unique variance which – also – contains measurement errors, the latter being per definition uncorrelated with the true scores). Thus, a standard assumption in SEM models is that the latent variables do not contain measurement error. As the latent variable scores are estimated by the estimation algorithm while the model is fitted, the scores are assumed to be free from measurement error. |
| A major problem with this paper is that many conclusions are written with causal language when the statistical analyses do not merit that. For example on page 10, there is a section header that says: “How does an art experience motivate support for climate policy?” Despite the identified relationship between the art experience and support for policy, we cannot know from this study anything about cause. As elaborated below, I believe that there are important variables that actually do impact policy support that are not measured or mentioned by the authors. This causal language comes up at several points, including the Discussion. Although the authors address this point in the Limitations, much of the language used earlier on is misleading. Similarly inappropriate causal language is used to explain the links between emotion and cognition, on which the authors are only speculating about directionality. | We went thoroughly through the paper and rephrased sentences which imply causality. We agree that with correlational data and a rather limited set of control variables we cannot make causal claims. |
| For Part 2 of the analysis (assessing an interaction), I would like to see more documentation of the model used, particularly all variables and coefficients, as well as error. | We added “S4 Table” to the supporting information to provide the requested information. In principle, this is a regression analysis with “reflection”, “environmental attitude” and their interaction term as predictors. To be consistent with the larger model, we included “gender” as control variable, but did not include it in the figure because it does not contribute significantly.  Also in this analysis, latent variable scores for “reflection” were used. As the outcome variable “support for climate policy” was ordered-categorical, we report the three thresholds in the table (see also the next comment for questions about the linking function).  Also in this analysis, the clustered / nested data structure was reflected in the analysis by adjusted standard errors. |
| I would like to know how the authors dealt with the fact that they had categorical data. How did the SEM accommodate this? | In cases of categorical dependent variables or indicators they are not connected to the latent variables or predictors through a standard linear regression link, but through a probit link function. MPLUS specifies the links accordingly when variables are defined as categorical (or ordered-categorical). |
| I see the dependent variable of support for climate policy as questionable. Support for policy is typically influenced by many factors not measured in this study, such as political preferences and worldview, especially when it comes to climate change. To say that the SEM in this study predicts climate policy is likely obscuring important predictive variables related to one’s political position. The lack of including a measure for political views undercuts the relationship between art viewing and climate policy support suggested by this study. | We agree that political preferences and worldviews are important predictors of support for climate policy. While these were not included in our study, we included environmental concern, which might be understood as a variable closely related to worldviews. As environmental concern, worldviews, and political preferences are often highly correlated, controlling for environmental concern covers – in our view – part of the potential influence of this variable cluster on climate policy. We added this thought to the discussion of limitations, though. |
