# Supplementary material for: Visual art inspired by climate change—An analysis of audience reactions to 37 artworks presented during 21st UN climate summit in Paris
Source: PLoS One. 2021 Feb 19;16(2):e0247331. doi: 10.1371/journal.pone.0247331 (PMC7894892; doi:10.1371/journal.pone.0247331)
Supplement: Attachment — Submitted filename: Response to Reviewers.docx [file pone.0247331.s006.docx]

# Response to Reviewer

Dear editor and reviewer,

We like to express our gratitude for your thoughtful inputs that have improved the manuscript. Please see the table below for all changes made to the manuscript based on your feedback.

| Reviewer’s comments | Actions taken |
| --- | --- |
| Thank you for taking such care in revising this manuscript. It is much clearer all around. I appreciate the new, detailed explanations of methods and the tempering of claims. The addition in the Limitations section is appreciated. The authors have responded to all of my feedback well. | Thank you for the appreciation. |
| First, I am slightly concerned about the unreliability of the Identifying with the Artist construct. While I agree with the authors that the unreliable multi-item variable is better than just a single item, my bigger question is what happens to the model when you drop the construct entirely? I recognize that you want to keep it in, as it aligns with your theory, but if it's unreliable, is it actually useful? I think the authors need to address this in some way--either further justify your keeping it, or show results with that construct dropped. | We followed the recommendation of the reviewer to drop “Identifying with the artist” from the analyses. This has some minor impacts on the model presented in figure 2, no impact on the model presented in figure 3, and no impact on the model presented in figure 4 (other than that the numbers slightly change). We have included the revised analyses in the paper now, updated figures and tables with the new numbers, and adapted the discussion sections accordingly. |
| Second, very minorly, there are a few typos. | We checked the manuscript one more time and fixed some typos. We hope that we have identified all. |
| Additionally, the new table (Table 4) could use some extra clarification about the Thresholds, as described in the authors' response to reviewers. | We added the explanations to table 4. |
